# A pilot and ex-vivo study of examination of endometrium tissue by catheter based optical coherence tomography

**DOI:** 10.1186/s12880-022-00890-7

**Published:** 2022-09-10

**Authors:** Bo Ding, Tao Jinyuan, Kuiyuan Tao, Zhenyang Ding, Shen Yang

**Affiliations:** 1 Department of Gynaecology and Obstetrics, The Affiliated Zhongda Hospital, Southeast University, Nanjing, 21009 China; 2 School of Precision Instruments and Opto-Electronics Engineering, Tianjin University, Tianjin, 300072 China

**Keywords:** Endometrium, Gynecology, Optical coherence tomography, Uterus

## Abstract

**Objective:**

This study aimed to distinguish ex-vivo normal and abnormal endometrium tissue samples histologically by catheter based optical coherence tomography (OCT).

**Methods:**

A total of 72 ex-vivo endometrium specimens were obtained from June 2018 to March 2021 and were imaged fresh after hysterectomy. The scanned region of endometrium was excised for histological examination and endometrium OCT images were precisely compared to corresponding histological images. Meanwhile endometrium OCT images were analyzed quantitatively with intensity of backscattered light in region of interest (ROI) and maximum penetration depth of the OCT signal. Blinded qualitative analysis on endometrium OCT images was performed by 2 assessors to determine accuracy rate and inter-rating reliability on the histopathological diagnosis.

**Results:**

OCT images were performed successfully in 72 endometrium specimens. Five endometrium specimens developed OCT interpretation criteria and the rest 67 endometrium specimens validated qualitatively and analyzed quantitatively. We defined an OCT criteria to distinguish normal endometrium and five different abnormal endometrium phases including proliferative endometrium, secretory phase endometrium, atrophic endometrium, endometrial hyperplasia with atypia and endometrial carcinoma based on OCT imaging features. The overall diagnosis accuracy achieved by the two assessors was 72.4% based on the OCT criteria. The inter-rater reliability between assessors on overall OCT images was substantial (Kendall τb of 0.720, *p* < 0.05). The changes in ROI minimum intensity, ROI maximum intensity, ROI average intensity and OCT signal maximum penetration depth of five different abnormal endometrium phases were significantly different (all *p* < 0.001). These parameters of endometrium carcinomas were significantly different from the other four endometrium phases (all *p* < 0.001).

**Conclusion:**

OCT has the advantage of noninvasive and rapid diagnosis, which can contribute to the diagnosis of endometrial cancer and will be an indispensable complement to traditional biopsy. Future studies in vivo with larger samples are needed to confirm this conclusion.

## Introduction

Currently, the diagnosis of endometrium carcinomas and its precursors requires histopathologic biopsies evaluation. The gold standard for diagnosis of endometrial cancer is histopathological examination, but it is invasive examination and time consuming. Traditional color Doppler ultrasound (US), computed tomography (CT) and magnetic resonance imaging (MRI) are widely used in clinical practice because of their non-invasive advantages, but they all have the disadvantage of insufficient tissue resolution, making it difficult to effectively distinguish normal tissues and abnormal lesions [1]. Moreover, CT examinations have the disadvantage of radiation, and MRI examinations are expensive, time-consuming and inconvenient to operate, which affect their practical application in the clinic [2].

Optical coherence tomography (OCT) is an optical imaging technique capable of building three-dimensional reflectance maps from minor signals within a scattering medium. Due to the independence of lateral and axial resolution [3, 4], OCT can achieve 10–20 micron accuracy and ultrahigh resolution, as is 10 times better than US [5, 6]. At the same time, OCT is non-invasive, which makes it difficult to be replaced by other methods [3–6].

OCT was first used in imaging the transparent tissue in the eye and had achieved wide application [7]. In recent years, OCT has been successfully used in the exploration of ophthalmology, cardiovascular, cerebrovascular and pulmonary diseases [8–11]. Moreover, studies have revealed that OCT had achieved satisfactory results in the detection of malignant tumors such as lung and gastrointestinal tumors [12]. In particular, it should be noted that in ovarian cancer and breast cancer, rather than judging the benign and malignant nature of surgical specimens, OCT has also been explored to determine the surgical margin without tumors and clarify the scope of surgical resection. This rapid diagnosis during surgery is expected to obtain a rapid diagnosis effect similar to the pathological diagnosis of frozen slices, and save more time than rapid pathology, which is incomparable to rapid intraoperative pathology [13–15].

In the field of endometrial examination, as early as 1998, Shakhova et al*.* had explored the morphology of the endometrium under OCT imaging technology [16]. Zhang et al*.* studied the OCT imaging characteristics of endometrial injury in rabbits and its correlation with infertility. These all shed light on the possible promise of OCT in endometrial imaging [17]. Later Law et al*.* [18] explored the imaging structure of the human endometrium, as well as the imaging status and characteristics of different lesions, suggesting that OCT had great potential for development in endometrial evaluation. However, it should be noted that the sample size of this research was relatively small, only 15 patients were included. The number of cases in each normal endometrial, endometrial hyperplasia with atypia and intimal cancer was very small, which made it difficult to obtain a very effective diagnosis. In addition, this study was conducted by a qualitative analysis of the intima, which had a more obvious subjectivity, affecting the robustness of the results. Therefore, a study with a large sample size and better resolution was needed to evaluate endometrial lesions by OCT, and at the same time to made a better assessment of the imaging characteristics of OCT.

In this study, we conducted a pilot and ex-vivo examination of endometrium tissues by catheter based OCT in ex-vivo uterine specimens from 72 total hysterectomy patients. The study revealed that OCT imaging had significant differences in the imaging characteristics of endometrial carcinoma and non-endometrial cancer in the histological structures of the endometrial epithelium and muscle layer, revealing the advantages of OCT imaging in the diagnosis of endometrial lesions and providing crucial value for the diagnosis of endometrial cancer.

## Methods

### Subjects and specimens

The study was approved by the independent ethics committee for clinical research of Zhongda Hospital, affiliated to Southeast University, Nanjing, China (clinical research ethics: 2020ZDSYLL093-P01). From June 2018 to June 2020 in our hospital, 72 endometrium specimens from 72 patients who underwent hysterectomy were included, including pathologically confirmed 32 proliferative endometrium, 6 mid-secretory phase endometrium, 11 atrophic endometrium, 7 endometrial hyperplasia with atypia and 16 endometrial carcinoma. All of the 72 informed consents from all subjects were obtained and the surgeries were carried out in accordance with relevant guidelines.

The inclusion criterias were: (1) Endometrium specimens were from patients with uterine fibroids, endometrial cancer, uterine prolapse and other diseases, who were recommended to underwent hysterectomy by the guidelines, or willing to remove the uterus, or those failed to receive conservative treatment. (2) Endometrium specimens were complete after hysterectomy. (3) There was no conflict between OCT imaging and histopathological diagnosis.

The exclusion criterias were: (1) Endometrium specimens were obtained from patients with partial hysterectomy. (2) Endometrium specimens could not be imaged due to uterine cavity deformation caused by multiple uterine fibroids. (3) Incomplete endometrium specimens could not be imaged caused by preoperative curettage. (4) Endometrium specimens were fragmented due to the use of a uterine manipulator.

### OCT imaging

**Fig. 1:**
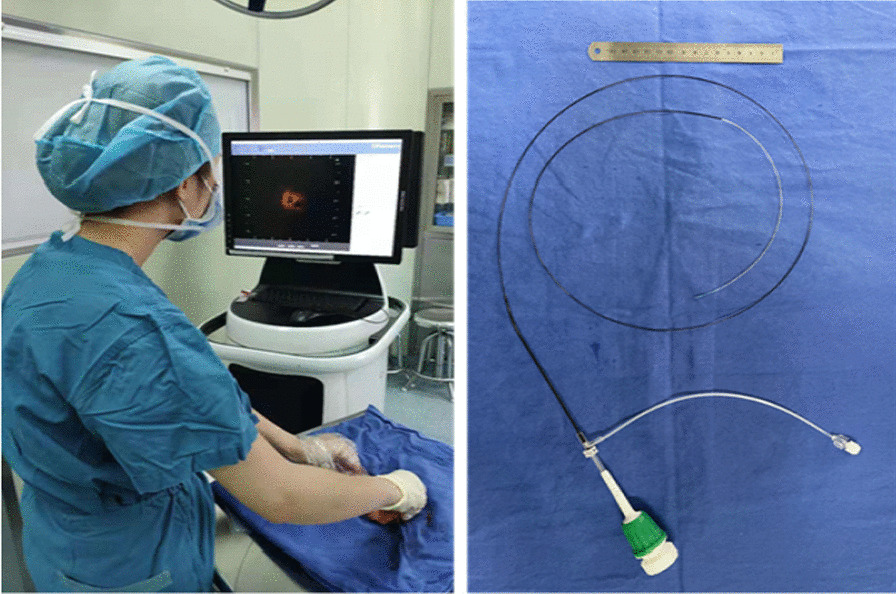
Photograph of OCT system used in the study and its catheter probe

Endometrium specimens were imaged fresh, within 0.5 h of hysterectomy. OCT imaging were performed using F-1 catheter based OCT system (Forssmann Medical Inc., Nanjing, China) and TY-1 imaging catheter (Forssmann Medical Inc., Nanjing, China). In order to balance the image resolution and depth of penetration, the F-1 system used a swept laser source of wavelength at 1310 nm which had a axial resolution around 15 µm and a lateral resolution around 20 µm. The signal penetration depth is defined as the depth at which the reduction in signal strength begins and ends with the appearance of background noise. The OCT imaging has a penetration depth of about 1–3 mm. Volumetric OCT images were performed by a 2.7 Fr (0.9 mm diameter) rotational scanning catheter probe or benchtop galvanometric scanner as previously described in Fig. 1. Rotational catheter probe was chosen as it was suitable for the imaging of tube-like hollow organs such as uterine cavity. The bandwidth of the OCT system is 100 nm and the power on the sample is approximately 10 mW. The A-scan rate of this OCT was up to 50 kHz. The images acquisition speed was 100 frames per second. 3D volumetric image acquisition required 2.7 s within one pullback.

The scanning window at the distal tip of the catheter was brought to the region of interest (ROI) to capture OCT image. The ROI was defined according to the catheter ruler. After specimen dissection, registration marks with an interval of 7–10 mm were placed in the ROI area with Indian ink to define the imaging window, which ensures “point-to-point” imaging.

The OCT imaging computer system had a designated program to display, save and output real-time OCT images. Longitudinal representations were prepared with ImageJ (National Institutes of Health, Bethesda, MD) and were displayed using a grayscale lookup table. Out-of-frame averaging (over two or three frames) was performed to reduce speckle noise. The image datasets were oriented to ensure that all tissues between the ink marks were visible for interpretation.

### Histology

OCT imaging were compared to pathological diagnosis as the gold standard. After OCT imaging, tissues were labeled and fixed in 10% formalin. The endometrium specimens at corresponding OCT scanned region were processed for histology, sectioned at 6 μm, stained with hematoxylin and eosin stain, and excised for standard pathological examination by two experienced pathologist according to standard histology procedures. The pathological diagnosis of the two pathologists was consistent.

### OCT interpretation criteria for endometrium

First five typical endometrium specimens including one proliferative endometrium, one secretory phase endometrium, one atrophic endometrium, one endometrial hyperplasia with atypia, and one endometrial carcinoma were selected to develop OCT image interpretation criteria. The OCT criteria were developed in conjunction with the corresponding histology to reflect the histological features, which was used to diagnose each type of endometrium histological status. The OCT criteria was formulated by OCT experts, pathologists and gynaecologist.

### Qualitative data analysis: blinded interpretation of OCT validation data

Two OCT assessors included a pathologist with expertise in gynecological cancer histology and a gynecological surgeon who routinely performs hysterectomy procedures. The two assessors, both with senior titles and experience, studied the characteristics of the pathological images and OCT images for each group of one case in the early stage, and after summing up their experience, they were asked to independently assess the remaining 65 OCT cases after randomization. Assessors had only access to the OCT interpretation criteria and were instructed to record a diagnosis of proliferative endometrium, mid-secretory phase endometrium, atrophic endometrium, endometrial hyperplasia with atypia or endometrial carcinoma for each case.

### Quantitative data analysis: intensity of backscattered light and maximum penetration depth of the OCT signal

We selected ROI minimum intensity, ROI maximum intensity and ROI average intensity (dB) and OCT signal maximum penetration depth to quantitatively compare the five endometrium phases. Signal penetration depth refered to the distance from the surface to the deepest region of the tissue in OCT images.

### Sample size

The quantitative analysis aimed to determine the inter-rater reliability for the pathology on endometrium under 5 endometrium phases as stated above. When the power and alpha were specified at 80% and 0.05, respectively, a minimum sample of 50 subjects was required to detect a minimum value of correlation coefficient of 0.4 [19].

## Statistics

All analyses were performed using SPSS 22 software (SPSS, Chicago, IL, USA). Continuous data are reported as the means ± standard deviations. The statistics were calculated for the blind validation assessment for each assessor. The accuracy and specificity of 5 different endometrium histological categories was calculated by comparison with the corresponding histologic diagnoses. The OCT inter- and intra-observer variability was quantified by the Cohen kappa test of concordance. Interrater-reliability was calculated for each histopathological diagnosis and expressed in the Kendall τ b correlation coefficient. Continuous variables were compared by using independent two-sample t test or analysis of variance (ANOVA). Differences were considered statistically significant when *p* < 0.05.

## Results

### Patient characteristics

The average age of patients ranged 28–65 years. Pathological diagnosis and indication of hysterectomy in the 72 specimens were summarized in Table 1. The endometrium specimens (n = 72) were divided into two groups: the criteria group (*n* = 5) and validation group (*n* = 67).

**Table 1:** Clinical characteristics of the recruited specimens

Pathological diagnosis of endometrium	Average age	Criteria group		Validation group		
		Number of specimens	Indication of hysterectomy	Number of specimens	Ratio (%)	Indication of hysterectomy
Proliferative endometrium	46.18 ± 6.66	1	Uterine fibroids: 1	31	46.3	Uterine fibroids: 31
Secretory Phase endometrium	45.69 ± 5.50	1	Uterine fibroids: 1	5	7.6	Uterine fibroids: 5
Atrophic endometrium	56.93 ± 5.59	1	Uterine prolapse: 1	10	14.9	Uterine fibroids: 4 uterine prolapse: 6
Endometrial hyperplasia with atypia	58.53 ± 8.26	1	Endometrial hyperplasia with atypia: 1	6	8.9	Endometrial hyperplasia with atypia: 6
Endometrial carcinoma	62.89 ± 8.26	1	Endometrial carcinoma: 1	15	22.3	Endometrial carcinoma: 5
Total		5		67		

### OCT imaging criteria for endometrium

#### Normal endometrium OCT imaging features

**Fig. 2:**
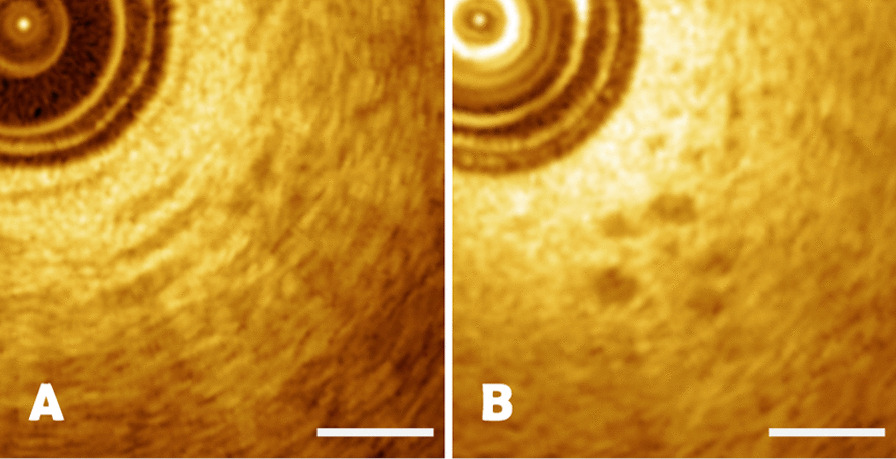
**A**–**B** OCT images of endometrial layer and endometrial cystic structure, with a scale length of 0.5 mm

The histopathologic features such as endometrium, myometrium and endometrial saccate structure such as endometrial glands could be acquired by OCT imaging. The endometrium was shown as a layer of high/medium density homogeneous signals on OCT images due to the normal cell density in endometrium (Fig. 2A). Endometrial saccate structure such as endometrial glands were shown as circular low-echo signs, which could be seen clearly particularly on the OCT images (Fig. 2B). The length of the scale bar was 0.5mm.

**Fig. 3:**
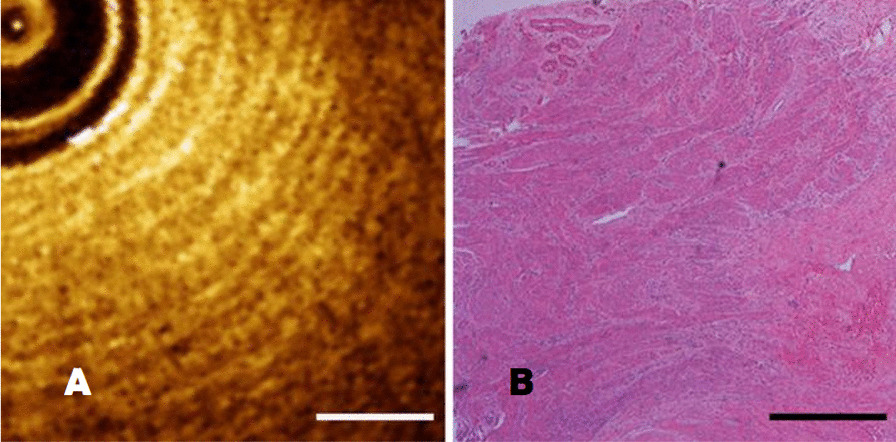
**A**–**B** The comparison of myometrium between OCT image and pathological section with a scale length of 0.5 mm

The myometrium was shown as heterogeneous intensity signal recognized as diffuse alternating bands of low and high density like rings or zebra stripes due to tissue morphology in myometrium (Fig. 3A). The histopathologic features of myometrium was shown in Fig. 3B, which were correlated with OCT images in the aspect of morphology, thickness and tissue structure. OCT image and histopathology were well matched with unique characteristics. The length of the scale bar was 0.5mm.

**Fig. 4:**
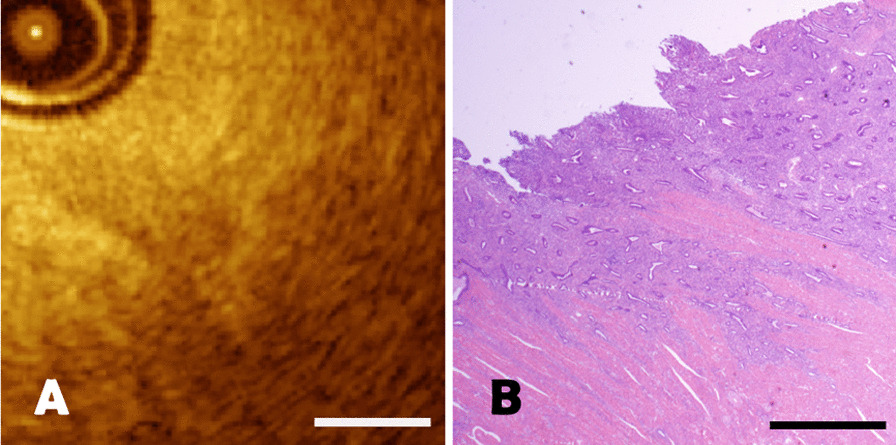
**A**–**B** The comparison of proliferative endometrium between OCT image and pathological section with a scale length of 0.5 mm

For proliferative endometrium, OCT imaging could distinguish functional endometrial layer and myometrium, as was shown in Fig. 4A. Endometrial glands present as tiny, dot-like, uneven, low-signal shadows, but were not specific. Histopathology section showed proliferative endometrium with multiple small glands and loose interstitium (Fig. 4B). The length of the scale bar was 0.5mm.

**Fig. 5:**
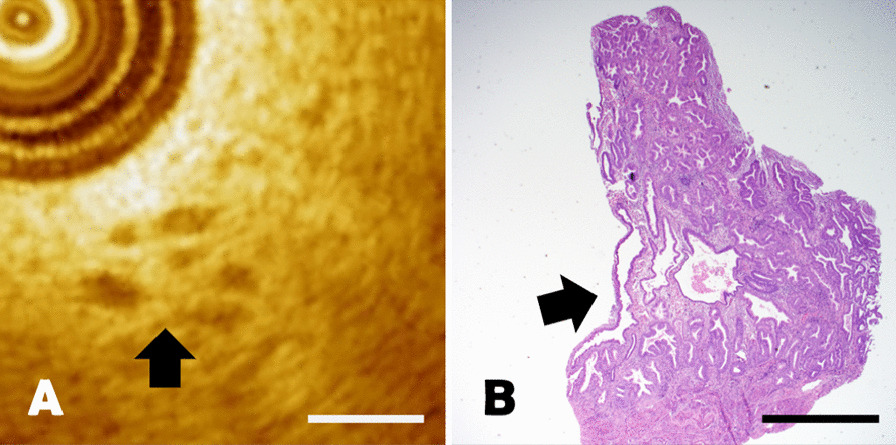
**A**–**B** The comparison of secretory endometrium between OCT image and pathological section. The black arrows represented the cystic structure of dilated gland, with a scale length of 0.5 mm

For secretory phase endometrium, the endometrial thickness was beyond the maximum depth of OCT imaging system. Myometrium was usually invisible. Multiple dilated glands with regular tortuosity identified were shown as a low-echo pattern in secretory endometrium area (Fig. 5A). Histopathology section manifested large and curved glands with dilated lumens and secretions in glandular lumen (Fig. 5B). The length of the scale bar was 0.5mm.

**Fig. 6:**
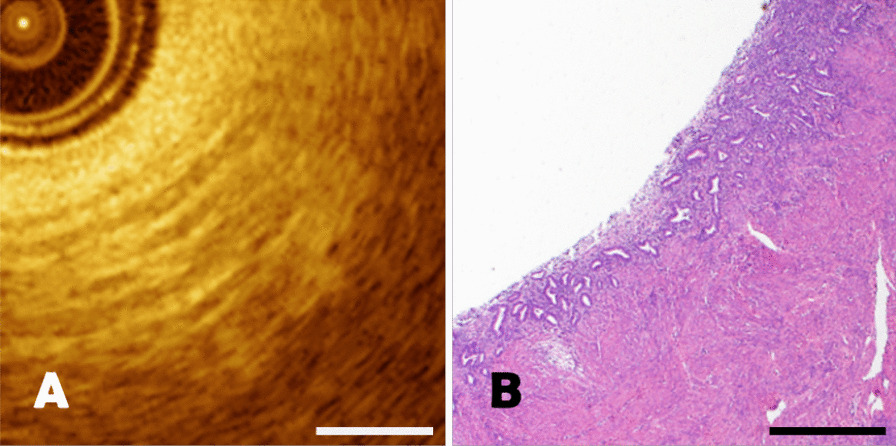
**A**–**B** The comparison of atrophic endometrium between OCT image and pathological section with a scale length of 0.5 mm

For atrophic endometrium, the most important feature was that the basal line and myometrium could be easily distinguished, and the glands were small (< 0.5 mm) or invisible. The OCT images of atrophic endometrium demonstrated high correspondence with pathological section (Fig. 6). The length of the scale bar was 0.5mm.

**Fig. 7:**
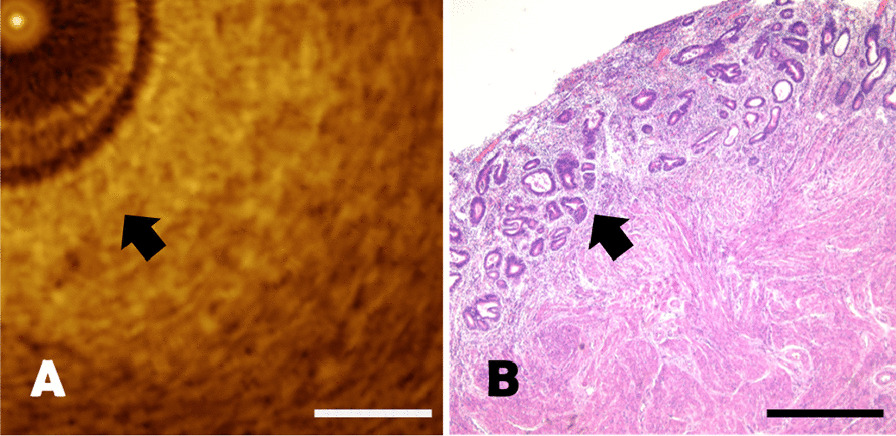
**A**–**B** The comparison of endometrial atypical hyperplasia between OCT image and pathological section. The black arrows represented the crowded small glands, with a scale length of 0.5 mm

It was difficult to distinguish endometrial hyperplasia with atypia from normal endometrium in OCT imaging. Endometrial hyperplasia with atypia in pathological sections was shown in Fig. 7B, there were marked crowded glands of various sizes with increased gland / stroma ratio. The variably crowded small glands could also be observed in OCT images as homogenous tissue structure (Fig. 7A). Whereas, atypical cells could not be identified on OCT imaging due to the limitation of spatial resolution. The length of the scale bar was 0.5mm.

**Fig. 8:**
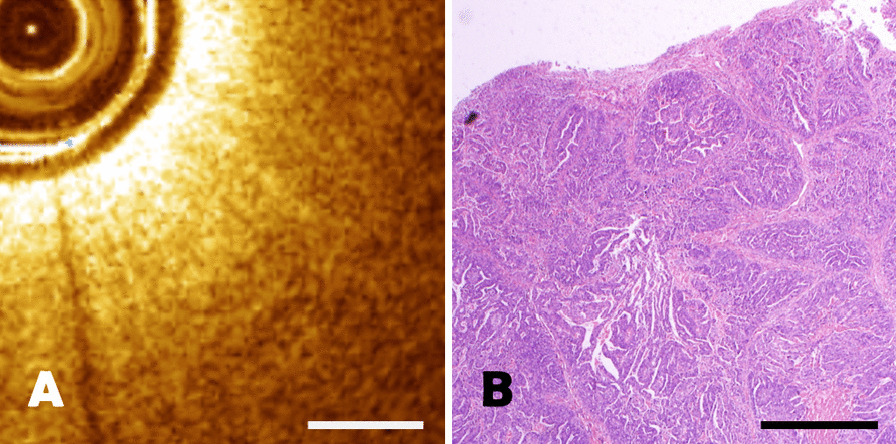
**A**–**B** The comparison of endometrial carcinoma between OCT image and pathological section with a scale length of 0.5 mm

For endometrial carcinoma, they were firstly shown as a layer of high-density heterogeneous signal likely due to high cell density, exhibited architectural disarray, and loss of normal structure on OCT imaging (Fig. 8A). Pathological section was characterized by incomplete glandular structure, missing local glandular structures and the formation of a solid tumor nest, at the same time, the epithelial cells showed immature differentiation, multilayered and polar disorders (Fig. 8B). The length of the scale bar was 0.5mm.

### Qualitative data analysis: blinded interpretation of OCT validation data

Two assessors applied a blinded interpretation of OCT validation data to distinguish five different abnormal endometrium phases based on OCT imaging features. The accuracy percentage was shown in the Table 2. The diagnosis average accuracy achieved by two OCT assessors was 72.4%.

**Table 2:** The diagnosis accuracy and inter-rater reliability between two assessors

	Assessor A accuracy			Assessor B accuracy			Kendall τb (*p* value)	Inter-rater reliability
	Correct/number of OCT image	Accuracy rate (%)	Specificity (%)	Correct/number of OCT image	Accuracy rate (%)	Specificit y(%)		
Proliferative endometrium	22/31	71.0	94.4	25/31	80.6	91.7	0.848 (*p* < 0.05)	Excellent
Secretory phase endometrium	3/5	60.0	88.1	4/5	80.0	89.6	0.705 (*p* < 0.05)	Substantial
Atrophic endometrium	8/10	80.8	95.5	7/10	70.0	94.0	0.697 (*p* < 0.05)	Substantial
Endometrial hyperplasia with atypia	2/6	33.3	91.8	1/6	16.6	91.8	0.404 (*p* < 0.05)	Moderate
Endometrial carcinoma	14/15	93.3	92.3	11/15	73.3	92.3	0.820 (*p* < 0.05)	Excellent
	49/67	73.1		48/67	71.6		Overall: 0.720 (*p* < 0.05)	Substantial

*p* < 0.05 means that the statistical difference was significant

The diagnostic accuracy of evaluator A and evaluator B for proliferative endometrium was 71.0% and 80.6%, respectively; diagnostic specificity was 94.4% and 91.7%, respectively. The diagnostic accuracy of secretory phase endometrium was 60.0% and 80.0%, respectively; diagnostic specificity was 88.1% and 89.6%, respectively. The accuracy of the diagnosis of atrophic endometrium was 80.0% and 70.0%, respectively; diagnostic specificity was 95.5% and 94.0%, respectively. The similar diagnostic accuracy of endometrial cancer was 73.1% and 71.6% respectively; diagnostic specificity was 91.8% and 91.8%, respectively. The diagnostic accuracy of endometrial hyperplasia was the lowest, 33.3% for evaluator A and 16.6% for evaluator B; diagnostic specificity was 92.3% and 92.3%, respectively.

Histopathological findings confirmed the validity of most of the evaluators' assessments. The inter-rater reliability (kappa statistic) of each different histological endometrium status ranged from 0.404 to 0.848. The overall OCT images were calculated and resulted in a Kendall τ b of 0.720, which meant a substantial agreement between assessors. Notably, there was a good agreement between the two assessors on the reliability of OCT images of malignant tumors (Kappa statistic 0.820).

### Quantitative data analysis: intensity of backscattered light and maximum penetration depth of the OCT signal

**Fig. 9:**
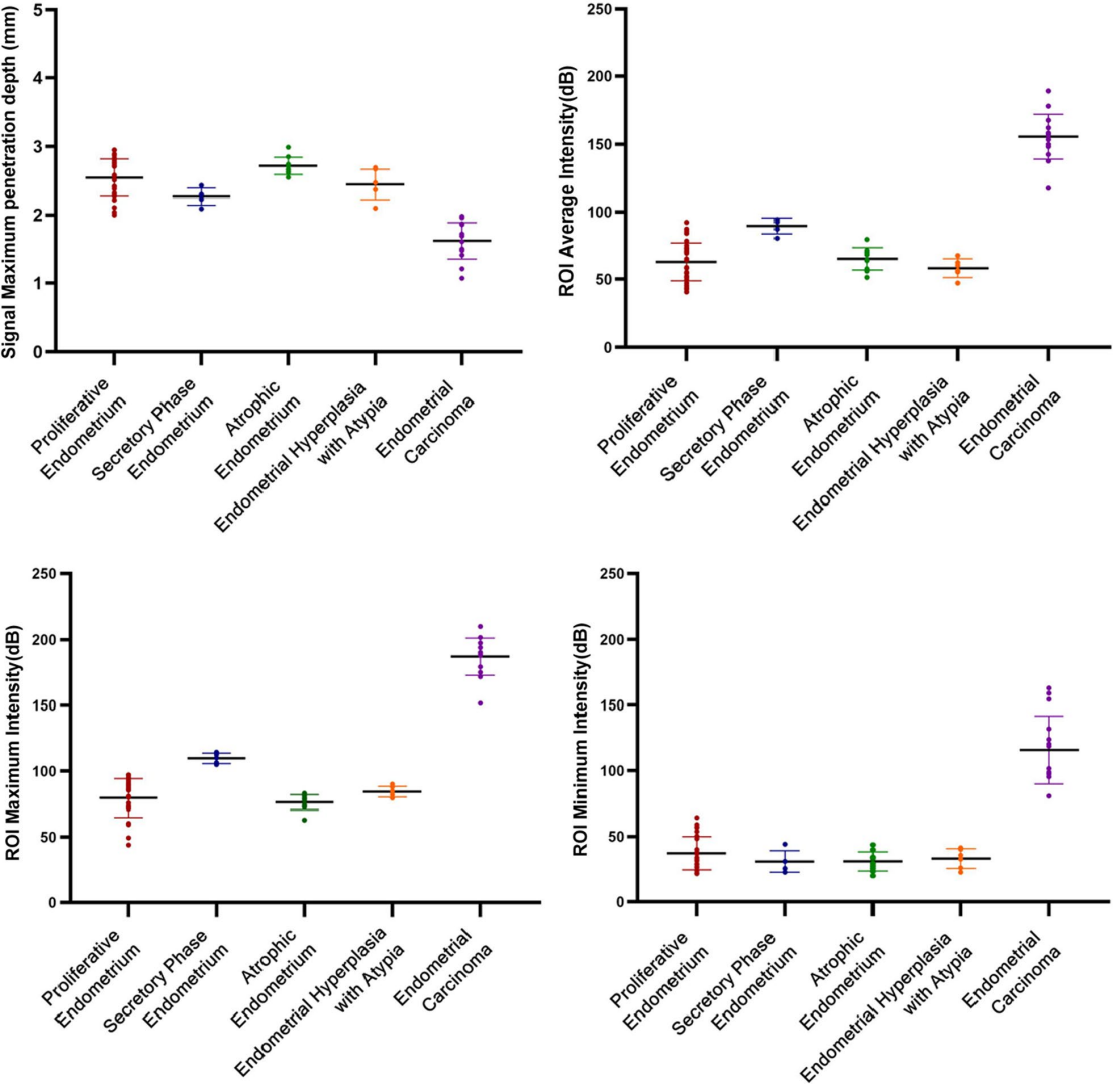
ROI backscattered light intensity (dB) and maximum penetration depth of optical signal (mm) under the surface of OCT image

ROI backscattered light intensity and maximum penetration depth of OCT signal were shown in Table 3. The changes in the ROI minimum intensity, the ROI maximum intensity, the ROI average intensity and the OCT signal maximum penetration depth of five different endometrium phases were significantly different (all *p* < 0.001). As shown in Fig. 9, the ROI minimum intensity, ROI maximum intensity, ROI average intensity, and maximum penetration depth of OCT imaging in endometrial cancer were significantly different from those in the other four endometrial stages (all *p* < 0.001). Specifically, the ROI minimum strength, ROI maximum strength and ROI average strength visibly increased compared with the other four groups, but the maximum penetration depth decreased significantly.

**Table 3:** ROI backscattered light intensity (dB) and maximum penetration depth of optical signal (mm) under the surface of OCT image

	Proliferative endometrium	Secretory Phase endometrium	Atrophic endometrium	Endometrial hyperplasia with atypia	Endometrial carcinoma	**F**	*p* value
ROI minimum intensity (dB)	37.23 ± 12.69	30.93 ± 8.20	31.02 ± 7.33	33.17 ± 7.59	115.87 ± 25.95	77.589	*p* < 0.001
ROI maximum intensity (dB)	79.67 ± 14.70	109.81 ± 4.02	76.40 ± 5.77	84.42 ± 4.01	187.05 ± 14.16	209.474	*p* < 0.001
ROI average intensity (dB)	62.90 ± 14.09	89.73 ± 5.85	65.23 ± 8.36	58.25 ± 6.95	155.62 ± 16.68	104.161	*p* < 0.001
OCT signal maximum penetration depth (mm)	2.54 ± 0.27	2.26 ± 0.12	2.71 ± 0.12	2.44 ± 0.21	1.62 ± 0.26	44.001	*p* < 0.001

*p* < 0.05 means that the statistical difference was significant

## Discussion

In this study, we defined a criteria for OCT imaging of endometrium tissues and found that OCT could be a novel technology with high accuracy and specificity to distinguish normal and abnormal endometrium tissues histologically ex vivo. Furthermore we developed five different endometrium status criteria based on these histopathologic characteristics and found that OCT images matched well with histopathology and had unique characteristics.

Based on this evaluation criterion, we developed and validated OCT interpretation criteria for different histological status of endometrium in a blinded assessment. The accuracy and specificity of both validation assessments and inter-rater reliability suggested the robustness of the results, which were similar to the accuracy of OCT criteria for esophageal [20] and cardiovascular disease in previous studies [21]. In this study, only five most common endometrium status were included in the criteria and validation assessments. This was done to keep the training manageable for our assessors and prevent confusion by educating them on too many entities in a short period of time. However, the differential diagnosis for different endometrium status was much more vast, given the array of potential etiologies for different endometrium status, it would be challenging to develop OCT criteria to distinguish all these entities with high enough accuracy, sensitivity and specificity to replace tissue biopsy.

At the same time, we found that OCT images of endometrial cancer showed their own characteristics. Firstly, because of the high cell density and loss of normal structure, endometrium carcinomas could exhibit a layer of high-density heterogeneous signal with architectural disarray on OCT images. Secondly, OCT imaging could identify the destructive growth of tumors, which were characterized by the disappearance of normal tissue boundaries and lack of the multilayered and ordered appearance of normal endometrial structures. Thirdly, due to the obvious optical attenuation and insufficient imaging penetration depths, the transmission of light declined gradually in the structures of endometrial cancer. These results were consistent with previous researches that cancer cells had a higher refractive index than normal cells [22]. Moreover, the structure of cancer tissue was often disorganized and typically characterized by variable cell sizes, abnormal shapes and enlarged nuclei, as resulting in different optical scattering properties and enabling OCT to distinguish differences effectively with high spatial resolution [13].

OCT was primarily used to visualize the morphology of tissue, subsequently, with the continuous improvement of OCT resolution, the current spatial resolution of OCT can reach 5 to 15 μm [23], and even sub-1-μm resolution has been demonstrated [24]. Therefore, OCT has been used to differentiate pathology in some circumstances at present [25–27]. Our study also confirmed that image features of endometrium carcinomas and other four endometrium phases differ significantly. At the same time, in the quantitative data analysis of OCT imaging, we found that the image characteristics of endometrial cancer differed significantly from the other four endometrial phases by calculating the ROI minimum intensity, ROI maximum intensity, ROI average intensity and OCT signal maximum penetration depth. This helps as an important addition to the diagnosis of endometrium carcinoma, while in the future is expected to replace the rapid freezing of pathology, reducing the waiting time for surgeons. In surgery, it is more critical to help surgeons determine the tumor-free incision margin to avoid impurity of resection, or blindly expand the scope of resection to cause damage to patients.

In this study we chosen 1310 nm wavelength, the most commonly used wavelength for studying the endometrium. This choice of wavelength was basically a balance between resolution and depth of penetration based on the characteristics of the biological tissue. We knew that if the wavelength of OCT was reduced, the higher the resolution of OCT imaging would be, the lower the depth of penetration would present [28]. In current clinical applications, the wavelength commonly used in ophthalmology was the 1060 nm band, and other biological tissues, such as blood vessels and bronchi, were commonly imaged in the 1310 nm band [29]. Such a long wavelength band was chosen based on the relatively thick nature of these luminal tissues, and a long wavelength band was beneficial in obtaining a relatively deep penetration depth. In this study, we have also chosen the most commonly used 1310 nm to study the endometrium, and this choice was based on the characteristics of the uterine cavity and endometrium. In the future we will also follow up with a shortwave-based programme to further study the tissue properties of the endometrium at higher resolution.

In recent years, ultra-high frequency ultrasound (UHFUS) is an emerging diagnostic technique with a wide range of applications in various clinical areas [30]. The range of frequencies between 30 and 100 MHz allows for high spatial resolution imaging of superficial structures, making UHFUS suitable for imaging skin, blood vessels, oral mucosa and small areas [30]. UHFUS has a higher penetration depth and a wider field of view for examination [31]. In the study of Sarkola et al., UHFUS resolution could reach 0.2 mm, and if the ultrasonic frequency was increased, the resolution could be up to 40 um [32, 33]. Nevertheless, the resolution of UHFUS was still worse than OCT, which utilized light wave imaging and the axial resolution could reach 10–20 um. OCT could image the composition of tissue surfaces as well as micro-structures, which gave OCT significant advantages [34]. At the same time, the UHFUS probe was not movable and required the catheter to be moved to get close to the lesion during imaging. In contrast, the OCT system was able to image a length of body cavity in seconds through rapid rotation and retraction techniques, which was an advantage that UHFUS could not match [35].

This study also had some limitations. First of all, it was conducted in the ex vivo setting to provide precisely correlated histology for accurate OCT feature characterization, which could not be quickly achieved in tumor tissue sections. However, it is important to note that tissue degradation and excess blood contamination from the surgical resection in ex vivo tissues can negatively impact OCT image quality and is not typically encountered in the in vivo setting. Therefore future in vivo studies will need to be performed to validate the findings of this ex vivo study. The second point to note is that although the overall accuracy obtained in this study was relatively high in the validated assessment of endometrial tissue, the results were not sufficient to support a complete replacement of OCT for traditional tissue biopsy. Because in OCT validation, many false-positive and false-negative cases needed to be diagnosed by the corresponding hematoxylin and eosin stain, depending on histological characteristics. In addition, with the advances in targeted therapy, physical tissue must be collected for molecular testing to determine whether tumors harbor mutations that would benefit from targeted therapy. This requirement precludes replacement of tissue biopsy with OCT and other in vivo optical biopsy techniques because, to date, they are unable to provide this essential mutational information.

## Conclusion

In summary, our study showed that OCT was able to clearly show the anatomy of the endometrium and could be used to determine the different phases of endometrial specimens. At the same time, we found that there was a good correlation between OCT images and pathological images, which revealed the clinical value of OCT in distinguishing benign and malignant lesions of the endometrium. OCT has the advantage of noninvasive and rapid diagnosis, which can contribute to the diagnosis of endometrial cancer and will be an indispensable complement to traditional biopsy. Future studies in vivo with larger samples are needed to confirm this conclusion.

## Data Availability

The datasets used and/or analysed during the current study available from the corresponding author on reasonable request.
